# Sulforaphane induces apoptosis and inhibits invasion in U251MG glioblastoma cells

**DOI:** 10.1186/s40064-016-1910-5

**Published:** 2016-02-29

**Authors:** Zhen Zhang, Chunliu Li, Li Shang, Yuejuan Zhang, Rong Zou, Yan Zhan, Benjun Bi

**Affiliations:** Yantai Affiliated Hospital of Binzhou Medical University, Yantai, 264100 China; Affiliated Hospital of Binzhou Medical University, Binzhou, China; Yantaishan Hospital, Yantai, China

**Keywords:** Sulforaphane, Apoptosis, Invasion, U251MG

## Abstract

In recent studies, sulforaphane (SFN) has been seen to demonstrate antioxidant and anti-tumor activities. In the present study, the viability inhibition effects of SFN in U251MG glioblastoma cells were analyzed by MTS. Morphology changes were observed by microscope. Apoptotic effects of SFN were evaluated by annexin V binding capacity with flow cytometric analysis. Invasion inhibition effects of SFN were tested by the invasion assay. The molecular mechanisms of apoptotic effects and invasion inhibition effects of SFN were detected by western blot and gelatin zymography. The results indicated that SFN has potent apoptotic effects and invasion inhibition effects against U251MG glioblastoma cells. These effects are both dose dependent. Taken together, SFN possessed apoptotic activity on U251MG cells indicated by increased annexin V-binding capacity, Bad, Bax, cytochrome C expression, and decreased Bcl-2 and survivin expressions. SFN inhibited invasion in U251MG cells via upregulation of E-cadherin and downregulation of MMP-2, MMP-9 and Galectin-3.

## Background

Glioblastoma, the most commonly primary central nervous system tumor, is one of the most devastating cancers with a 15 months median survival and a 8–26 % 2-year survival rate (Hassler et al. 2014; Messaoudi et al. 2015; Nagasawa et al. 2012). Despite research efforts, effective therapies for glioblastoma are limited. Chemotherapy, as one of the most important therapies, has been proved to improve survival in glioblastoma (Spiegel et al. 2007; Stupp et al. 2009; Yang et al. 2014). However, rapid emergence of tumor resistance and side effects of current chemotherapeutic agents lowered the therapeutic effects and limited application (Chamberlain 2010; Chen et al. 2013). Thus, novel anti-glioblastoma drugs with fewer side-effects and greater therapeutic efficiency are strongly demanded.

The poor prognosis of glioblastoma is ascribed to the rapid growth rate and the high invasiveness (Alifieris and Trafalis 2015; Onishi et al. 2013; Wurth et al. 2014). Therefore, an efficient chemotherapeutic agent must be able to inhibit both growth and invasion of glioblastoma cells. Besides, the agent must have low toxicity to normal cells and have no tumor resistance。

At present, natural products are the major sources of available anticancer drugs. Phytochemicals obtained from vegetables, fruits, teas, and medicinal plants have been extensively investigated for their anti-cancer activities due to their safety, low toxicity, and general availability (Pratheeshkumar et al. 2012). Identifying their active ingredients and researching the molecular mechanisms of such ingredients is regarded as an attractive approach for drug development. Sulforaphane (SFN), first identified in broccoli sprouts and present at high concentrations in most cruciferous vegetables, has been shown to be effective in cancer treatment. Studies showed that SFN could induce apoptosis, inhibit progression and metastasis of many kinds of cancer cells (Chen et al. 2015). And, there is an increasing need for new drugs to treat high-risk cancer patients with a higher selectivity for cancer cells and lower toxicity to normal cells. SFN has a safe cytotoxicity profile towards the normal cells thus it can be utilized in the development of safe cancer treatment strategies (Hussain et al. 2013; Kallifatidis et al. 2011). In addition, sulforaphane can overcome the chemoresistance of cancer cells (Lan et al. 2015; Pastorek et al. 2015).

Despite a great number of studies describing the chemopreventive and chemotherapeutic properties of sulforaphane in cancers, there is little information on the properties of sulforaphane in glioblastoma. In the present study, we examined the effects of SFN on U251MG glioblastoma cells. Our study aimed to provide a novel potential therapeutic agent for the treatment of glioblastoma. Our results indicated that SFN was effective in inhibiting the tumor growth and infiltration of U251MG cells.

## Results

### SFN inhibited viability and changed cell morphology of U251MG glioblastoma cells

**Fig. 1:**
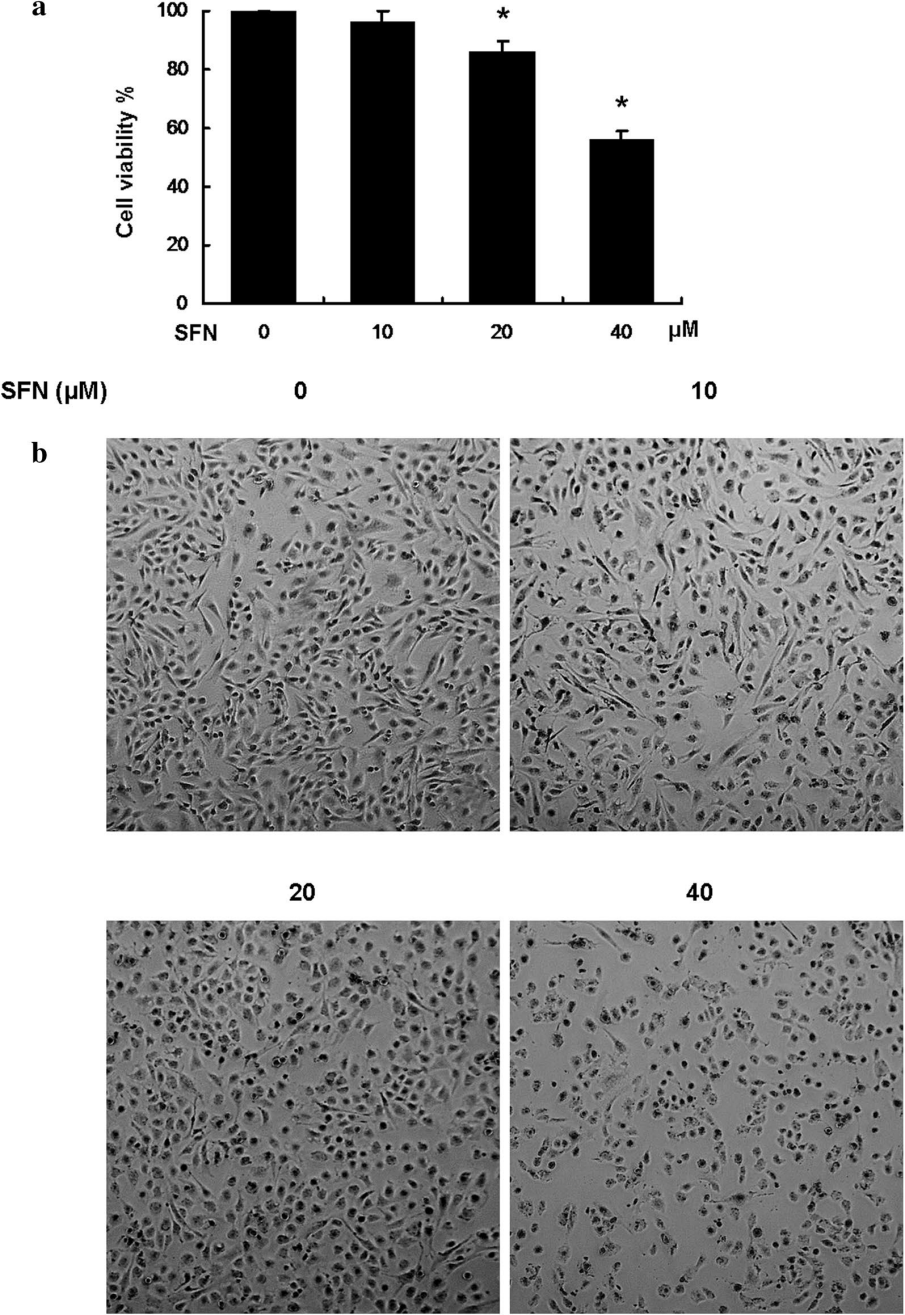
SFN inhibited viability and changed cell morphology of U251MG cells. **a** U251MG cell lines were treated with 0, 10, 20 and 40 µM SFN for 24 h. Cell viability was examined using MTS assay. Results were expressed as a percentage of control, which was considered as 100 %. Data are presented as the mean ± SD of three independent experiments. **b** Morphological changes of U251MG cells treated with different concentrations of SFN for 24 h (magnification, ×40). The cells were evidently reduced in size and their margins were unclear compared with the control group

**Fig. 2:**
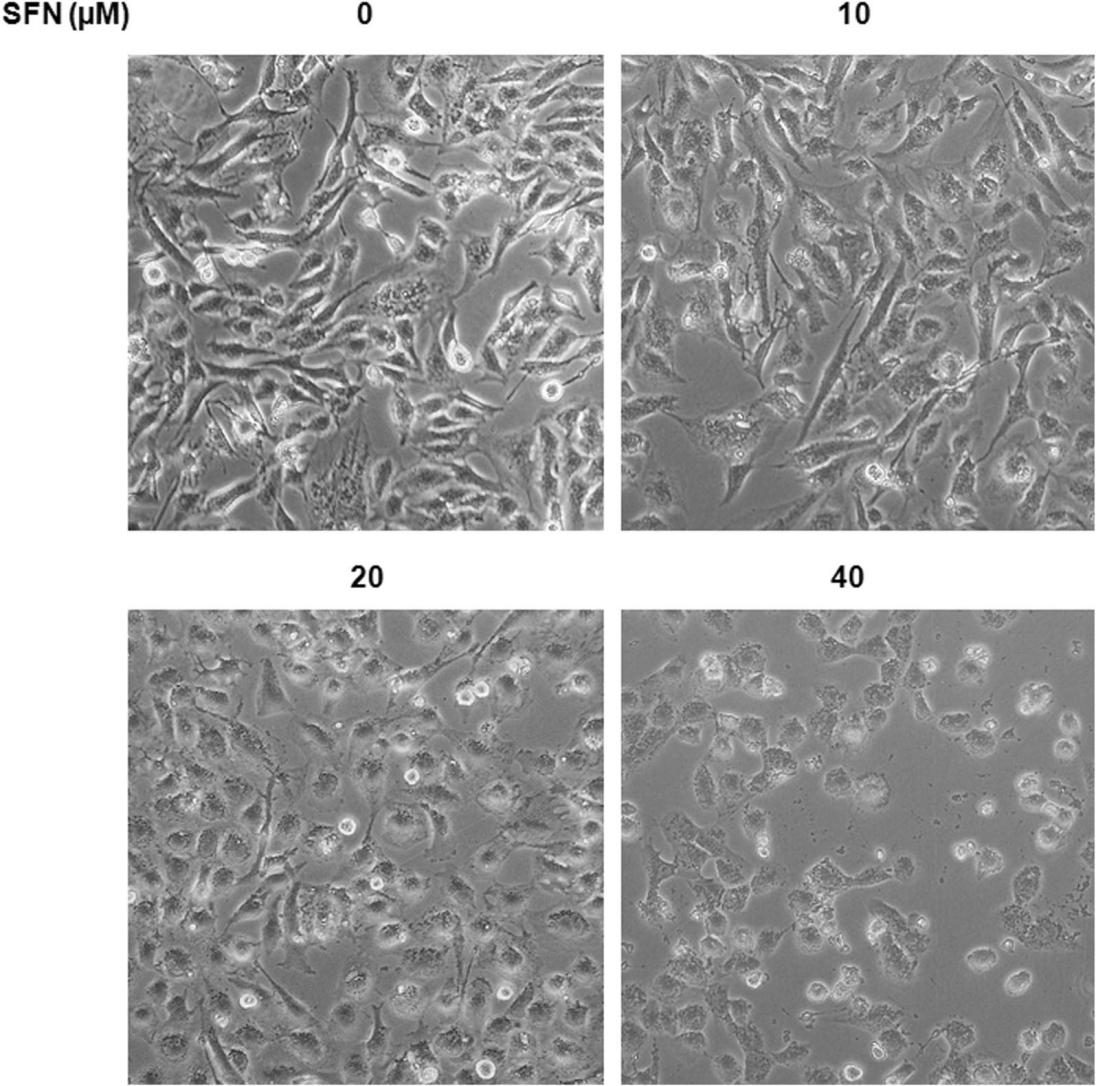
SFN inhibited viability and changed cell morphology of U251MG cells. Morphological changes of U251MG cells treated with different concentrations of SFN for 24 h (magnification, ×100). The cells were evidently reduced in size and their margins were unclear compared with the control group

The viability of U251MG cells was measured by MTS assay. U251MG cells were treated with the increasing doses of SFN (0, 10, 20 and 40 µM) for 24 h. MTS assay showed that SFN significantly reduced the viability of U251MG cells in a dose dependant manner (Fig. 1a). And we found that SFN obviously changed cell morphology. Following 24 h of SFN incubation, U251MG cells demonstrated reduction of pseudopodia, a rounded shape, shrinkage, nuclear condensation and fragmentation (Figs. 1b, 2). These results indicated the potential apoptosis inducing effect and invasion inhibiting effect of SFN in U251MG cells for further analysis.

### SFN induced apoptosis in U251MG glioblastoma cells

**Fig. 3:**
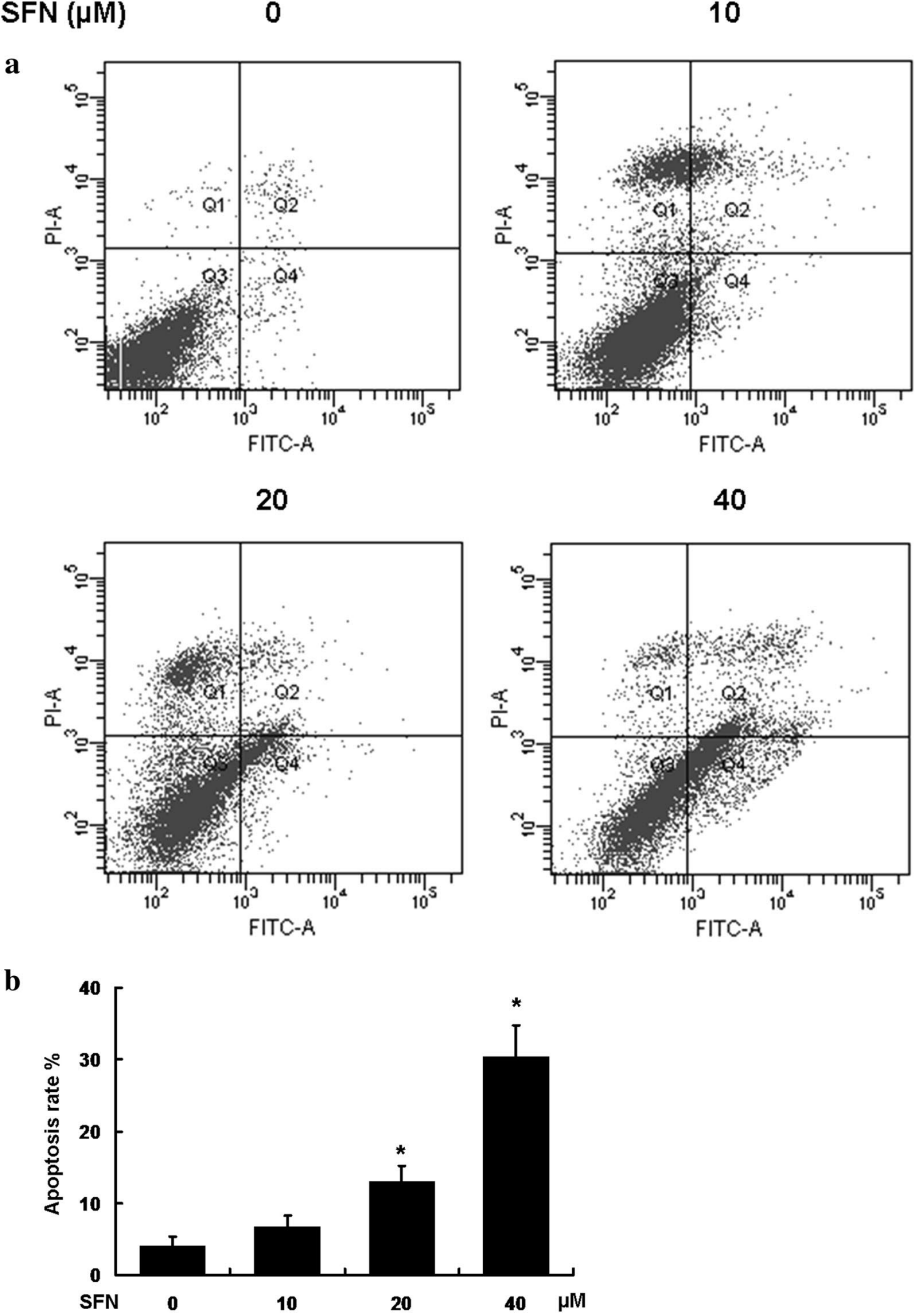
SFN induced apoptosis. U251MG cells treated with 0, 10, 20, 40 µM SFN for 24 h. **a** The cells were stained with annexin V and PI. The percentages of apoptosis were based on the cells present in the LR and UR quadrants. Total apoptosis percentage, indicating that SFN induced apoptosis in a dose dependent manner. **b** Statistical analysis of total apoptosis percentage. At 10 µM SFN, no signifiant differences were observed compared with the control group (P > 0.05). At 20 and 40 µM, P < 0.05 versus the control group. *PI* propidium iodide, *LR* lower right, *UR* upper right

Based on the finding that SFN was associated with cell apoptosis, we further studied the potential influence of SFN on U251MG cell apoptosis. After treated with SFN for 24 h, the apoptosis ratios were detected by flow cytometry. As shown in Fig. 3, the apoptosis ratios of the control group was 4.03 ± 1.31 %. Respectively, the apoptosis ratios of the U251MG cells treated with 10, 20, 40 µM SFN were 6.70 ± 1.54, 13.03 ± 2.11, and 30.40 ± 4.33 %. When treated with 10 µM SFN, the percentage of total apoptosis increased, but the difference was not statistically significant compared with the untreated group (P > 0.05). Treated with ≥20 µM SFN, the apoptosis ratio was dramatically increased in a dose-dependent manner (*P < 0.05).

### SFN induced apoptosis in U251MG via regulating related proteins

**Fig. 4:**
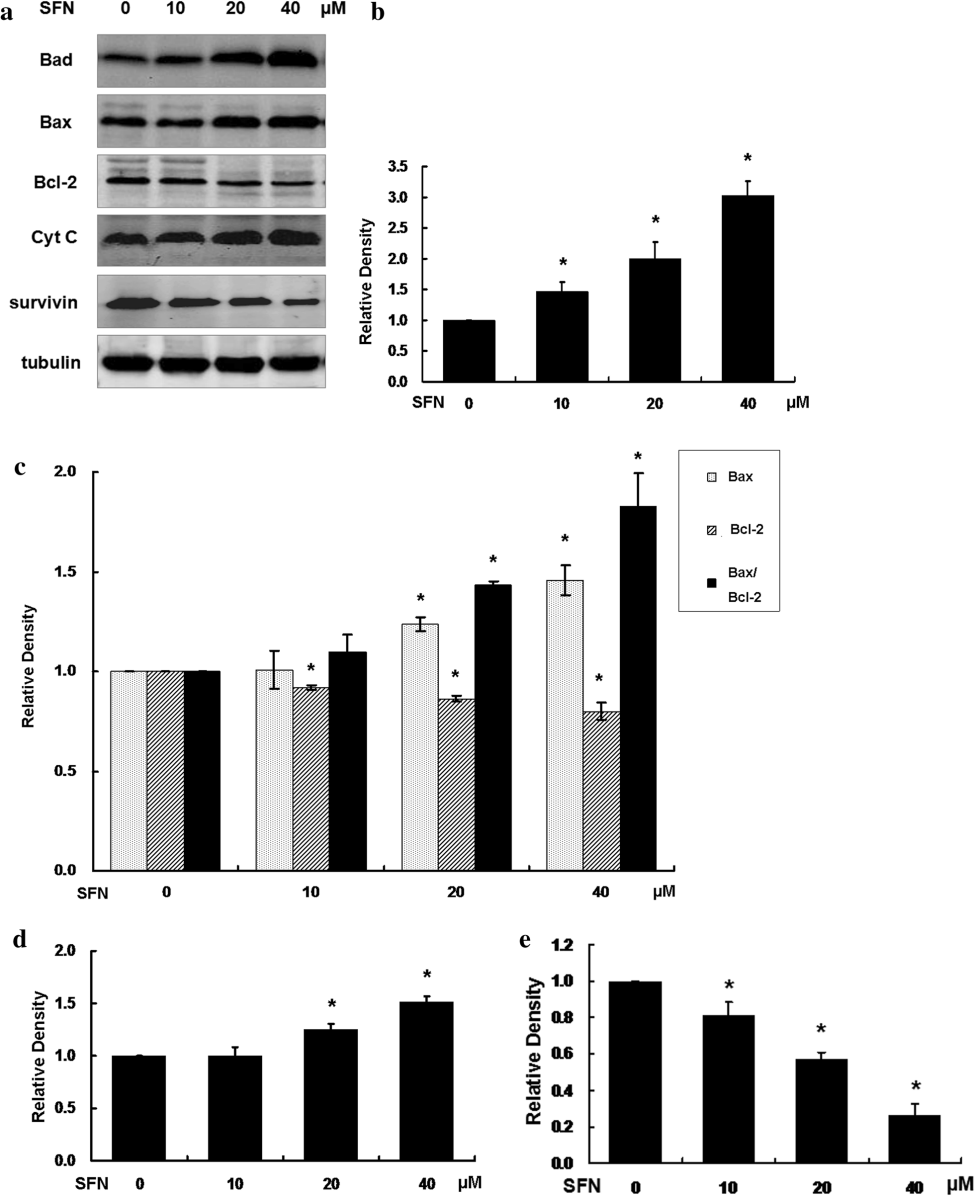
SFN regulated expressions of Bcl-2, Bax, Bad, cytochrome C and survivin. **a** Expression of apoptosis-associated proteins in U251MG glioblastoma cells treated with SFN and the expression levels of Bcl-2, Bax, Bad, and cytochrome C were detected by western blot analysis using tubulin as a control. **b** Statistical analysis of Bad expression. **c** Statistical analysis of Bax, Bcl-2 expression and the ratio of Bax/Bcl-2. **d** Statistical analysis of cytochrome C expression. **e** Statistical analysis of survivin expression

Western blot analysis revealed that the expression levels of Bcl-2, Bax, Bad, cytochrome C in SFN-treated cells were upregulated, while the Bcl-2 and survivin expression levels were downregulated when compared with the control group (Fig. 4a). (b) Statistical analysis of Bax, Bcl-2 expression and the ratio of Bax/Bcl-2. At 10 µM SFN, no signifiant differences were observed compared with the control group (P > 0.05). At 20 and 40 µM, P < 0.05 versus the control group. (c) Statistical analysis of Bad expression. SFN upregulated Bad expression in a dose dependent manner. (d) Statistical analysis of cytochrome C expression. At 20 and 40 µM, P < 0.05 versus the control group. (e) Statistical analysis of survivin expression. The Bax, Bad, cytochrome C, Bcl-2 and survivin expression levels were affected in a dose-dependent manner in the U251MG cells.

### SFN inhibited invasion in U251MG glioblastoma cells

**Fig. 5:**
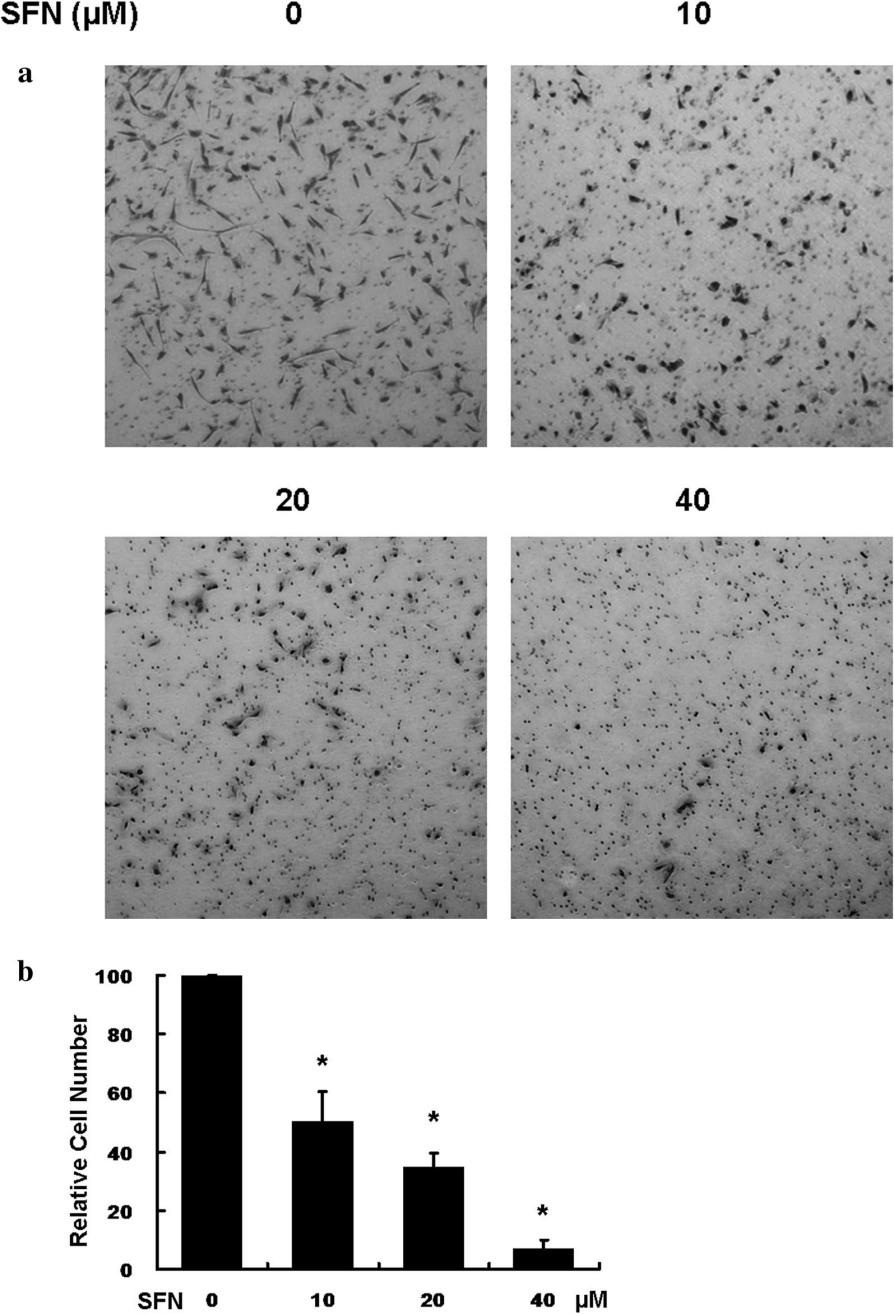
SFN inhibited invasion. **a** U251MG glioblastoma cells were seeded in transwells pre-coated with matrigel and were treated with 0, 10, 20, and 40 μM SFN for 24 h, respectively. Then, randomly chosen at least 5 fields were photographed (×40), and the number of cells that invaded into the lower surface was calculated as a percentage of invasion. **b** Statistical analysis of total invasion percentage. The *bar chart* showed that the number of invasive cells was significantly lower in SFN-treated U251MG cells than that of control cells (*P < 0.05)

To determine whether SFN weakens the cell invasion potential, transwell matrigel invasion assays were conducted. The cells invading through the matrigel were counted. The results showed that the cell numbers in the following groups (10, 20, 40 µM) treated with SFN for 24 h were significantly decreased versus control cells (0 µM) following a dose-dependent manner (Fig. 5). Clearly, SFN attenuated the cell motility ability significantly.

### SFN inhibited invasion ability in U251MG via influencing E-cadherin, Galectin-3 and MMPs

**Fig. 6:**
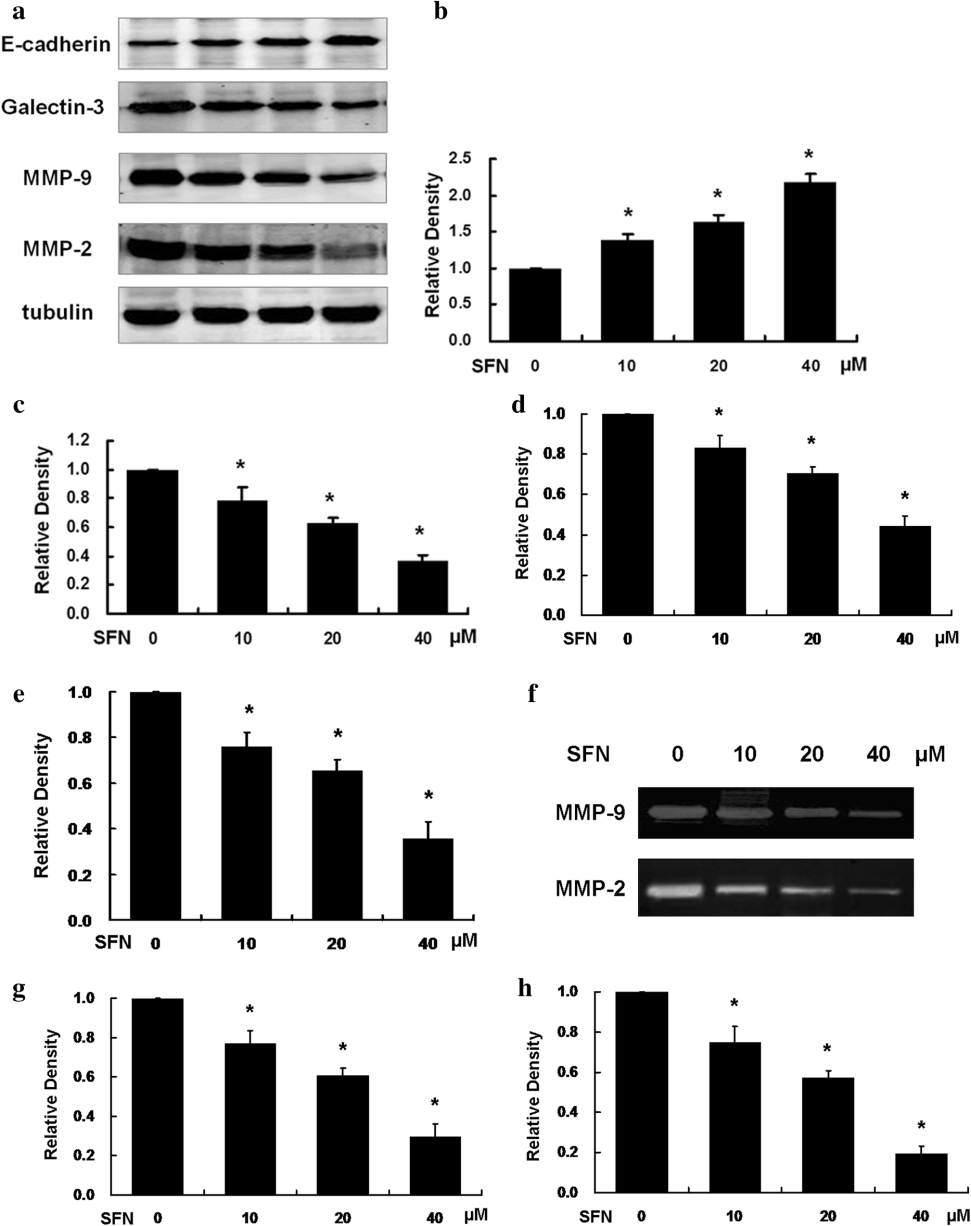
SFN regulated E-cadherin, Galectin-3, MMP-2 and MMP-9. **a** Expression of invasion-associated proteins in U251MG cells treated with SFN and **b** statistical analysis of E-cadherin expression. **c** Statistical analysis of Galectin-3 expression. **d** Statistical analysis of MMP-9 expression. **e** Statistical analysis of MMP-2 expression. **f** Activities of invasion-associated proteins in U251MG cells treated with SFN and **g** statistical analysis of MMP-9 activity. **h** Statistical analysis of MMP-2 activity. The expression levels of E-cadherin, Galectin-3, MMP-2 and MMP-9 were detected by western blot analysis using tubulin as a control. The activities of MMP-2, MMP-9 were detected by gelatine zymography. The results are representative of at least three independent experiments

E-cadherin, Galectin-3, MMP-2 and MMP-9 play important roles in cellular invasion. The results of western blot showed that the protein levels of E-cadherin was increased, Galectin-3, MMP-2 and MMP-9 were decreased significantly after SFN treatment (Fig. 6a). To determine whether the activities of MMP-2 and MMP-9 were also influenced by SFN, we employed Gelatine zymography. As demonstrated in Fig. 6f, samples from SFN-treated cells had darker bands than the control cells at both 72 and 92 kDa. That indicates SFN is able to inhibit the activities of MMP-2 and MMP-9.

## Discussion

Sulforaphane (SFN), which is purified from cruciferae, has been demonstrated to possess a number of pharmacological properties and is one of the most promising agents for the treatment of various cancer types (Clarke et al. 2008). The present study investigated the influence of SFN on glioblastoma cells.

The results of the MTS assay confirmed that SFN effectively reduced the growth of U251MG cells in a dose-dependent manner. Furthermore, at increasing concentrations of SFN, the cells became rounder and marcider, which was the morphological characteristics of apoptosis.

Subsequently, the percentages of apoptosis were distinguished using an annexin V and PI method by flow cytometric analysis. Promoting tumor cell apoptosis has been recognized as one of most important methods towards tumor treatment (Feng et al. 2011; Lino and Merlo 2011). We found that SFN induced apoptosis of U251MG cells in an increasing trend. Upon treatment with 10 µM SFN, the percentage of total apoptosis increased, but the difference was not statistically significant compared with the untreated group (P > 0.05). Therefore, this SFN concentration was hypothesized to be relative low and unable to trigger further cell apoptosis that would be detected by annexin V and PI. By contrast, at 20 and 40 µM SFN, the apoptosis rates were identified to be significantly different compared with the control group (P < 0.05). In addition, certain studies have previously reported that SFN induced cell apoptosis in different cancer cell lines (Conzatti et al. 2014; Gupta et al. 2014; Huang et al. 2012), which is consistent with the results of our study.

The uncontrolled growth and diffuse infiltration of tumor cells are the major cause of recurrence and death. Effective anti-glioblastoma therapy should not only inhibit tumor cell growth, but also prevent invasion of cancer cells (Ranjit et al. 2015). We further investigated the effects of SFN on the invasion ability of U251MG glioblastoma cells. The invasion assay showed that SFN inhibited invasion ability of U251MG cells. The results conformed with studies which demonstrated that SFN inhibited invasion in different cancer cell lines (Lee et al. 2013; Lenzi et al. 2014; Shan et al. 2013). In short, SFN is a potential chemopreventive agent for glioblastoma.

However, the molecular mechanisms vary depending on cell lines (Gupta et al. 2014). In the present study, we found that SFN could inhibit U251MG glioblastoma cells, but the molecular mechanisms through which SFN affects glioblastoma cells remain unclear. In order to clarify the exact mechanisms of apoptosis and invasion inhibition effects in U251MG glioblastoma cells, we investigated the influence of SFN on the crucial proteins associated with apoptosis and invasion.

Mitochondria are central integrators and transducers of proapoptotic signals in apoptosis (Carloni et al. 2012; Deighton et al. 2014). Bcl-2 family proteins play a crucial role in the mitochondrial apoptosis pathway. To a large extent, the sensitivity of cells to apoptotic stimuli, depends on the proportion of Bax protein to Bcl-2 protein in the cells. When the proportion increases, the cell apoptosis rate rises. Bad is a negative regulation of Bax/Bcl-2 heterodimer. It replaces Bax in a concentration dependent manner, and the free Bax form the homodimer (Hattori et al. 2000; Hutcheson et al. 2015; Yang et al. 1995). Formation of Bax/Bax homodimers lead to loss of mitochondrial outer membrane integrity and cytochrome C release into the cytoplasm, and then triggers the occurrence of the whole apoptotic events. That means Bad mediates apoptosis by regulating the ratio of homodimer to heterodimer of Bax.

We studied apoptosis mechanism of SFN on glioblastoma through the comprehensive expression of Bcl-2, Bax, Bad and cytochrome C. According to the results of the present study, SFN downregulated the expression of Bcl-2, upregulated the expression of Bax, Bad, and the proportion of Bax/Bcl-2 proteins, which indicated that Bcl-2 family proteins are involved in the SFN triggered apoptosis in U251MG cells. Furthermore, caspases are key factors in the execution of apoptosis. Caspase-3 is the most critical apoptosis performer in the downstream of cascade, and the activation of caspases is largely dependent on the release of cytochrome C (Xie et al. 2015; Yi et al. 2015). The present results demonstrated that the expression levels of cytochrome C were elevated following SFN treatment. It is most likely that SFN induces apoptosis by elevating their upstream signal Bax-to-Bcl-2 ratio resulting in cytochrome C escape to the cytoplasm where cytochrome C interacts with caspases signaling pathways. Meanwhile, SFN increased the expression of Bad which upregulated the homodimers of Bax. Analysis by synthesis, we concluded that the proapoptosis signaling way of SFN in U251MG glioblastoma cells was SFN–Bad–Bax/Bcl-2-cytochrome C-caspases.

Besides, survivin is the smallest and strongest member of the inhibitor of apoptosis protein (IAP) family that is selectively overexpressed in most common types of human cancers, and has been implicated in the control of cell division, inhibition of apoptosis, and tumor cell resistance to certain anticancer agents. Survivin is one of the most specific cancer antigens identified to date and blocks apoptosis via antagonizing caspases, It is expressed in over 80 % of glioblastomas, but rarely detectable in normal adult tissues. In human gliomas, the overexpression of survivin was closely associated with uncontrolled cell proliferation and the inhibition of apoptosis, high levels of survivin expression revealed to be correlated with a poor prognosis. We found that SFN downregulated survivin in U251MG cells. Therefore, survivin is an important target molecule of SFN.

In order to clarify the exact mechanisms of invasion inhibiting effects of SFN on U251MG glioblastoma cells, the expression and activity of key invasion proteins were examined.

Tumor cell invasion into the basement membrane is the fundamental steps that mediate the dissemination of cells from the primary site to distant secondary sites. In this process, matrix metalloproteinases (MMPs) play crucial roles. Studies showed that MMPs degraded extracellular matrix proteins to create space for invading glioblastoma cells, and activate signal transduction cascades that promote invasion. Several studies address the fact that MMPs especially MMP-2 and MMP-9 overexpressed in glioblastoma cells compared with normal brain tissue (Chintala et al. 1999; Nakada et al. 2003). And the inhibition of MMPs could significantly reduce the invasiveness of glioblastoma cells (Coniglio and Segall 2013). In our study, secretion of MMP-2 and MMP-9 from U251MG cells was decreased after administering SFN. We also found SFN reduced the activity of MMP-2 and MMP-9 in U251MG cells. That’s a main reason for invasion inhibition of SFN in U251MG glioblastoma cells.

E-cadherin was reduced or lost in the majority of glioma tissues. E-cadherin plays a key role in cancer cell invasion. Galectin-3, one of the most important factors in cell invasion process, was reported to be modulated by MMPs, which plays a crucial role in glioma cell invasion. Studies showed that high levels of Galectin-3 expressed in high grade glioma (Le Mercier et al. 2010). These studies are consistent with our results that SFN downregulated Galectin-3 and inhibited invasion in U251MG cells. Galectin-3 is an important target molecule of SFN in glioblastoma cells invasion progress.

## Conclusions

**Fig. 7:**
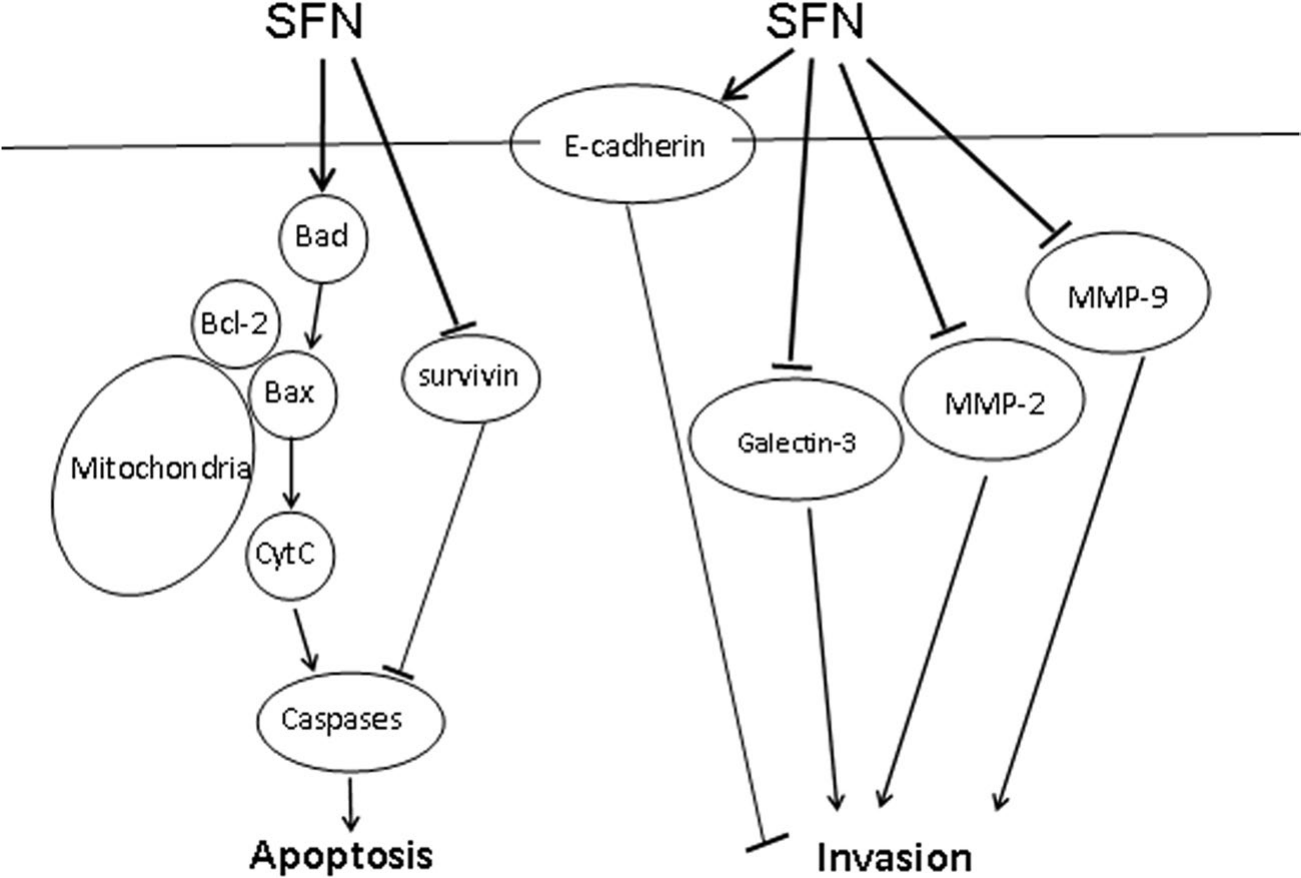
Signaling pathway of SFN in U251MG cells

In conclusion, SFN has potent apoptosis inducing effects and invasion inhibiting effects against U251 glioblastoma cells. These effects are dose dependent. The most likely mechanisms are the induction of apoptosis by Bad–Bax/Bcl-2-cytochrome C signaling pathway and survivin, and inhibition of invasion by influencing E-cadherin, Galectin-3, MMP-2 and MMP-9 (Fig. 7). This study suggests that SFN may be a suitable candidate for glioblastoma treatment (Ciftci et al. 2015). However, to the best of our knowledge, numerous mechanisms are involved in SFN-induced apoptosis and invasion inhibition, further research is required to elucidate these.

## Methods

### Cell lines and culture

The glioblastoma cell lines, U251MG, were obtained from ATCC (Manassas, VA, USA). The cells were cultivated in DMEM/HIGH glucose culture medium containing 10 % fetal bovine serum (FBS), 100 U/ml penicillin and 100 U/ml streptomycin. Cells were grown at 37 °C in a humidified incubator containing 5 % CO2. The medium was refreshed every 2–3 days. Cells were trypsinized by trypsin. The cells in the logarithmic growth phase were used to conduct the experiments described as follows.

### Reagents

Culture media were purchased from Hyclone (Logan, Utah, USA), FBS and penicillin–streptomycin were acquired from Invitrogen (Carlsbad, CA, USA). MMP-9 antibody was purchased from Cell Signaling Technology, Inc (Shanghai, China). MMP-2 antibody was supplied by Santa Cruz Biotechnology (Santa Cruz, CA, USA). Antibodies against Bax, Bad, Bcl-2, cytochrome C and α-tubulin were purchased from Proteintech Group, Inc (Chicago, USA). d, l-Sulforaphane (SFN) was acquired from Sigma (St Louis, MO, USA). MTS assay kit was purchased from Promega (Madison, USA). Transwell plates for invasion assay were bought from BD Biosciences (Bedford, MA, USA). BCA protein assay kit was obtained from Thermo Scientific Pierce Protein Research Products (Rockford, IL., USA). Annexin V-FITC Apoptosis Assay Kit was acquired from Nanjing KeyGEN Biotech (Nanjing, China).

### MTS

Cells were seeded into 96-well culture plates at the rate of 5000 cells/well overnight. Different cell wells were treated with 0, 10, 20 and 40 µM SFN for 24 h. 20 µl pre-warmed MTS reagent was added to each of the wells for an additional 1 h following treatment. The absorbance values were measured at 490 nm by a BioTek^®^ microplate reader (SynergyTM, HT, USA). In the absence of cells, background absorbance of the medium was subtracted. Results were expressed as a percentage of control (0 µM SFN), which was considered as 100 %. Each assay was performed in triplicate, and the results were expressed as the mean ± SD.

### Morphological observation

U251MG cells were seeded and grown in 6-well culture plates until they reached 70 % confluence. Then SFN was administered at concentrations of 0,10, 20 and 40 µM and cells were cultured for 24 h. Morphological changes were observed by an phase-contrast microscope at ×40 and ×100 magnification (Leica, Germany) connected to a digital camera (Olympus, Japan).

### Flow cytometry analysis of apoptotic cells

Apoptosis was detected using annexin V-FITC/propidium iodide (PI) staining followed by flow cytometry. After treated with SFN for 24 h, cells were collected and centrifuged at 1000 rpm for 5 min. Then, cells were washed twice with cold PBS to remove excessive medium. Next, the cells were re-suspended at a concentration of 1 × 10^6^ cells/ml in binding buffer and incubated with annexin V-FITC and PI at room temperature for 15 min in the dark. A total of at least 10,000 cells were collected and analyzed by flow cytometry (FACSAria, BD, USA).

### Western blot

Samples were separated on SDS-PAGE gels and then transferred to nitrocellulose membranes. Membranes were incubated overnight at 4 °C with primary antibodies. Membranes were washed with TBS-T, and then incubated with secondary antibody. The same membranes were stripped and reprobed with α-tubulin antibody for equal loading and normalization.

### Cell invasion assays

Cell invasion was performed using BD BioCoat Matrigel invasion chambers (BD Biosciences, 8 µm) pre-coated with BD Matrigel matrix. The assay insert plates were prepared by rehydrating the BD matrigel with 300 ml pre-warmed serum-free medium at room temperature for 30 min. 1 × 10^5^ of U251MG cells treated with different concentrations of SFN (0, 10, 20, 40 µM) suspended in 300 µl medium without FBS were added separately onto the apical chambers, and 500 µl medium with 10 % fetal bovine serum was added in the lower chambers. After the cells were grown for 24 h, the cells on the apical chambers were removed with a cotton swab. The invaded cells (or pseudopodia) on the bottom of the chamber were fixed with 100 % methanol for 20 min at −20 °C, then stained with 0.5 % crystal violet solution at room temperature for 20 min. After moving away the crystal violet solution, the cells were rinsed with distilled water until no excess dye was viewed. The invaded cells or pseudopodia were counted from 5 randomly selected areas, the images were photographed by camera with a Leica DM IRB microscope at ×40 magnification.

### Gelatin zymography

Gelatin zymography has been widely used to measure the activity of matrix metalloproteinases MMP-2 (gelatinase A) or MMP-9 (gelatinase B). Cells were incubated in serum-free medium for 24 h with different concentrations of SFN (0, 10, 20, 40 µM) treatment. Medium with secreted MMP-2 and MMP-9 proteins was collected from an equal number of cells and was centrifuged at 2000 rpm for 10 min to remove cellular debris. Then we collected the supernatant and mixed it with equal amount of sample buffer for loading in the gel. The supernatants were separated at 4 °C in a 10 % SDS polyacrylamide gel containing 1 mg/ml gelatin as a protease substrate. In the process of electrophoresis, SDS is reversibly combined with MMPs of samples to destroy hydrogen bond and hydrophobic bond, and MMPs can’t decompose gelatin. After electrophoresis, in order to eluate SDS, the gels were washed in renaturing buffer (pH 7.5, 2.5 % Triton X-100) for 30 min, 2 times; then incubated in fresh developing buffer (50 mM Tris–HCl, pH 7.5, 0.2 M NaCl, 10 mM CaCl_2_, 0.02 % NaN_3_) at 37 °C for 42 h to renature gelatinases. The gel was incubated with staining buffer [0.5 % Coomassie Blue R-250, 30 % methanol, and 10 % acetic acid)] and then destained with washing buffer (50 % methanol, 10 % acetic acid), compared with marker, the zymography band at the specific location (molecular weight 62) was confirmed as active MMP-2, at the specific location (molecular weight 92) was confirmed as MMP-9.

### Statistical analysis

Statistical analysis was performed using Statistical Package for Social Science (SPSS) SPSS 18.0. Data were expressed as mean ± SD and were evaluated by Student’s *t* test or one-way ANOVA. Multiple between-group comparisons were performed using the Student–Newman–Keuls (S–N–K) method. Every experiment was repeated at least three times. A P value <0.05 was considered to be statistically significant.

## Abbreviations

SFN: sulforaphane
MMP: metalloproteinase
ATCC: American Type Culture Collection
PI: propidium iodide
FBS: fetal bovine serum
